# A multi-national report on methods for institutional credentialing for spine radiosurgery

**DOI:** 10.1186/1748-717X-8-158

**Published:** 2013-06-27

**Authors:** Peter C Gerszten, Arjun Sahgal, Jason P Sheehan, Ronald Kersh, Stephanie Chen, John C Flickinger, Mubina Quader, Daniel Fahim, Inga Grills, John H Shin, Brian Winey, Kevin Oh, Reinhart A Sweeney, Matthias Guckenberger

**Affiliations:** 1 University of Pittsburgh Medical Center, Pittsburgh, USA; 2 University of Toronto, Toronto, ON, Canada; 3 University of Virginia, Charlottesville, VA, USA; 4 Riverside Medical Center, Newport News, VA, USA; 5 Oakland University William Beaumont School of Medicine, Royal Oak, MI, USA; 6 Massachusetts General Hospital, Boston, MA, USA; 7 University Hospital Würzburg, Wuerzburg, Germany; 8 Department of Neurological Surgery, Presbyterian University Hospital, Suite B-400, 200 Lothrop St, Pittsburgh, PA 15213, USA

**Keywords:** Spine Radiosurgery, Stereotactic Body Radiotherapy, Credentialing, Spine Tumors

## Abstract

**Background:**

Stereotactic body radiotherapy and radiosurgery are rapidly emerging treatment options for both malignant and benign spine tumors. Proper institutional credentialing by physicians and medical physicists as well as other personnel is important for the safe and effective adoption of spine radiosurgery. This article describes the methods for institutional credentialing for spine radiosurgery at seven highly experienced international institutions.

**Methods:**

All institutions (n = 7) are members of the Elekta Spine Radiosurgery Research Consortium and have a dedicated research and clinical focus on image-guided spine radiosurgery. A questionnaire consisting of 24 items covering various aspects of institutional credentialing for spine radiosurgery was completed by all seven institutions.

**Results:**

Close agreement was observed in most aspects of spine radiosurgery credentialing at each institution. A formal credentialing process was believed to be important for the implementation of a new spine radiosurgery program, for patient safety and clinical outcomes. One institution has a written policy specific for spine radiosurgery credentialing, but all have an undocumented credentialing system in place. All institutions rely upon an in-house proctoring system for the training of both physicians and medical physicists. Four institutions require physicians and medical physicists to attend corporate sponsored training. Two of these 4 institutions also require attendance at a non-corporate sponsored academic society radiosurgery course. Corporate as well as non-corporate sponsored training were believed to be complimentary and both important for training. In 5 centers, all cases must be reviewed at a multidisciplinary conference prior to radiosurgery treatment. At 3 centers, neurosurgeons are not required to be involved in all cases if there is no evidence for instability or spinal cord compression. Backup physicians and physicists are required at only 1 institution, but all institutions have more than one specialist trained to perform spine radiosurgery. All centers believed that credentialing should also be device specific, and all believed that professional societies should formulate guidelines for institutions on the requirements for spine radiosurgery credentialing. Finally, in 4 institutions radiation therapists were required to attend corporate-sponsored device specific training for credentialing, and in only 1 institution were radiation therapists required to also attend academic society training for credentialing.

**Conclusions:**

This study represents the first multi-national report of the current practice of institutional credentialing for spine radiosurgery. Key methodologies for safe implementation and credentialing of spine radiosurgery have been identified. There is strong agreement among experienced centers that credentialing is an important component of the safe and effective implementation of a spine radiosurgery program.

## Background

Stereotactic body radiotherapy (SBRT) and stereotactic radiosurgery (SRS) are rapidly emerging treatment options for both malignant and benign spine tumors [1-23]. While there has been a long history and great experience with frame based intracranial radiosurgery using a variety of technologies at institutions around the world, there is far less experience with the adoption of these frameless extracranial radiosurgery technologies. The term SBRT implies high-dose-per-fraction radiation (typically > 5 Gy per fraction) delivered to an image-guided target in 1 to 5 fractions by using conformal radiation techniques [24]. SBRT for the spine is technically demanding because it often requires near-rigid body immobilization, sophisticated treatment planning allowing for sharp dose gradients, and imaging guidance to ensure that the dose is delivered accurately.

In conventional fractionated radiotherapy that does not employ image-guidance, the number of fractions can range anywhere from 5 to 25, and the individual dose per fraction is smaller than with SBRT [24]. Compared to conventional fractionated radiotherapy, the steep dose gradients and tight margins achieved with spine SBRT have an even greater probability to lead to detrimental consequences via either a reduced tumor control or an increase in normal tissue toxicity [25]. It is for this reason that there has been an increased interest and awareness in the steps necessary to safely and effectively develop an institution’s spine radiosurgery program.

Quality assurance (QA) is a key component for modern image-based radiotherapy, and many formal guidelines and recommendations have been published on this subject [26,27]. Many of these guidelines have been set forth by national and international professional organizations. However, the majority of such work in QA for radiosurgery has focused on the successful implementation of QA in the context of multi-centered clinical trials [25,28-31]. Others have published practice guidelines endorsed by governing organizations for the performance of SBRT to serve as an educational tool designed to assist practitioners in providing appropriate care for patients [32]. With respect to spine SRS, it is worthy to mention the recent scope of practice guidelines endorsed by the Canadian Association of Radiation Oncology as they were focused on spine, lung and liver SBRT [26]. Within this document are recommendations as to the role of the radiation oncologist, suggestions as to training recommendations and an overview of quality assurance measures for departments to consider when initiating such a program. While most of these guidelines cover a wide range of issues related to the delivery of extracranial radiosurgery, they generally fail to address the issue of individual institutional credentialing of personnel involved in a spine radiosurgery program.

Radiosurgery is a new and important treatment option for spinal neoplasms. Proper credentialing by physicians, medical physicists, and radiation therapists is important for the safe and effective adoption of spine radiosurgery and ultimately may be associated with improved patient outcomes. The purpose of this study is to provide a comprehensive overview of the current requirements at experienced institutions for spine radiosurgery credentialing.

## Methods

The Elekta Spine Radiosurgery Research Consortium (ESRRC) is a research group consisting of seven international institutions, all with a research and clinical focus on image-guided high precision radiotherapy in general and spine radiosurgery in particular. Six of seven institutions are academic hospitals (University Hospital Wuerzburg [UHW], Wuerzburg, Germany; Princess Margaret Hospital [PMH], Toronto, Canada; University of Pittsburgh Medical Center [UPMC], Pittsburgh, US; University of Virginia Medical Center [UVAMC], Charlottesville, US, Massachusetts General Hospital [MGH], Boston, US, Oakland University William Beaumont School of Medicine [WBH], Royal Oak, US), and one is a private radiotherapy center fully specialized in image-guided radiosurgery (Riverside Regional Medical Center [RSMC], Newport News, US]. All seven institutions are highly experienced in treating spine and paraspinal tumors using image-guided radiosurgery, and all academic centers have contributed to the recent technical and clinical progress in spine radiosurgery [33].

For better comparability of methods and clinical outcome, the consortium was established such that all centers use identical equipment for delivery of spine radiosurgery. Treatment is planned for a high-resolution multi-leaf collimation with a 4 mm leaf width on the Elekta Axesse linac (Elekta Axesse, Elekta AB, Stockholm, Sweden), daily volumetric image-guidance is performed with cone-beam technology (Elekta XVI, Elekta AB, Stockholm, Sweden), set-up errors are corrected in six degrees of freedom (HexaPOD, Elekta AB, Stockholm, Sweden) and all patients are thoroughly immobilized in the BodyFIX system (BodyFIX, Elekta AB, Stockholm, Sweden).

This consortium previously published in great detail its methods for both treatment planning, including treatment planning systems, as well as treatment techniques [33]. This manuscript further described a direct comparison of techniques between the seven institutions. Spine radiosurgery was used at these centers to treat a variety of benign, primary malignant, as well as metastatic tumors. The specific indications for spine radiosurgery employed by this consortium has been described previously and is beyond the scope of the current manuscript. Indications for radiosurgery included as a primary treatment modality, for recurrence or progression after conventional fractionated radiotherapy, as an adjunct to open surgery, and for lesions not amenable to open surgery.

A 24 item questionnaire was established covering all major aspects of spine radiosurgery credentialing and completed by all members of the ESRRC. Questions regarding credentialing fell under the following broad categories: (1) policies and procedures, (2) training requirements, (3) surgeon involvement, (4) the role of industry and professional organizations, and finally (5) perceptions regarding the importance of credentialing requirements. The questionnaires were answered by the responsible physician from each institution and reflect their current practice of spine radiosurgery.

### Complete study questionnaire

The following represents all questions included in the study questionnaire:

At your institution, is there currently a written policy in place regarding the credentialing process for spine radiosurgery?

If there is no written policy, is there currently an unwritten policy that is followed for performing spine radiosurgery?

What formal requirements are there for training medical physicists who perform spine radiosurgery?

What formal requirements are there for training physicians who performs spine radiosurgery?

Must medical physicists attend a corporate sponsored training course to perform spine radiosurgery?

Must physicians attend a corporate sponsored training course to perform spine radiosurgery?

Must medical physicists attend a formal training course sponsored by a professional organization in order to perform spine radiosurgery (e.g. AAPM, ASTRO, ESTRO)?

Must physicians attend a formal training course sponsored by a professional organization in order to perform spine radiosrugery (e.g. ASTRO, ESTRO, AANS)?

Are all spine radiosurgery cases reviewed at some multi-disciplinary conference beforehand?

Is a neurosurgeon involved in all spine radiosurgery cases?

Under what circumstances is a neurosurgeon not involved in a spine radiosurgery case?

Does your institution require that a “backup” physicist be trained and credentialed for spine radiosurgery?

Does your institution require that involved physicians (radiation oncologists and neurosurgeons) have a “backup” specialist be trained and credentialed for spine radiosurgery?

If your institution currently does not have a formal credentialing process for spine radiosurgery, do you intend to create one?

How important do you feel a credentialing process for spine radiosurgery is for each of these categories?

Patient safety?

Patient clinical outcomes?

Hospital management?

The development of a new spine SRS program?

Should the manufacturers of radiosurgery equipment assist new users in the development of institutional credentialing?

Is spine radiosurgery education for users’ better delivered in the format of industry sponsored educations courses that are specific to one technology or by radiosurgery courses sponsored by professional organizations such as ASTRO, ESTRO, AANS, CNS, or AAPM?

Should credentialing for spine radiosurgery at an institution be technology specific or should it be sufficient for all technologies capable of radiosurgery within an institution and leave it to the individual clinician to ensure that they have a sufficient knowledge base to use other technologies if they so desire?

Is there credentialing for all imaging methods used in radiosurgery planning and follow-up ?

Are the involved radiation therapists formally trained and credentialed by industry for spine radiosurgery on specific machines?

Are the involved radiation therapists credentialed by appropriate professional organizations for performing spine radiosurgery?

## Results

The questionnaire was completed by all 7 institutions and responses were recorded, tabulated, and analyzed. The responses were reviewed by all members of the ESRRC. In general, close agreement was observed in most aspects of spine radiosurgery credentialing at each institution. In order to better categorize and evaluate the responses, the following technique was employed. Agreement by at least six members of the group was considered as “strong agreement”. Agreement by at least four members of the group (greater than half the group) was considered as “some agreement”.

### Areas of strong agreement

There were six areas of strong agreement among institutions for spine radiosurgery credentialing. There areas were the following:

»Formal credentialing process for all physicians, physicists, and therapists.

»Need for a written policy specific for spine radiosurgery credentialing.

»Reliance upon in-house proctoring system for physicians, physicists, and therapists.

»Credentialing should be device specific.

»Professional organizations develop guidelines for institutions credentialing requirements.

»Importance of credentialing for safety and clinical outcomes.

### Areas of some agreement

There were four areas of some agreement among institutions for spine radiosurgery credentialing. There areas were the following:

»Radiation oncologists, neurosurgeons, physicists, and therapists should be required to attend corporate-sponsored device specific training.

»Requirement for professional organization training.

»Corporate and non-corporate training complement one another; both are necessary.

»Requirement to have more than one specialist trained to perform spine radiosurgery as a back up.

### Policies and procedures

As of the time of the survey response completion, only one of the institutions had a written policy specific for spine radiosurgery credentialing. That policy was specific for neurosurgeons only, and did not apply to radiation oncologists or physicists. All centers have an undocumented credentialing system in place that mirrored their intracranial and general body radiosurgery systems.

### Training requirements

All institutions relied upon an in-house proctoring system for the training of both physicians and medical physicists. Most centers require a proctoring of at least 10 patients for credentialing. Medical physicists were required to complete a formal device specific training course at 4 centers and proctoring at 6 centers. Physicians are required to complete a corporate sponsored course at 4 centers. Only in the European center were physicians also required to complete a training course in extracranial radiosurgery provided outside of their institution.

Formal training in the techniques of multimodality imaging and image registration in the region of the spine for target contouring and treatment image guidance was highly variable. In 2 centers, formal training is required for physicians in imaging methods used in radiosurgery treatment planning. Physicists were not required to undergo specific training on image registration nor small field dosimetry. In 3 centers, plans are in place to formalize and document their credentialing process for spine radiosurgery.

All centers felt that credentialing should be device specific. Finally, in 4 institutions radiation therapists were required to attend corporate-sponsored device specific training for credentialing, and in only 1 institution were radiation therapists required to also attend professional society training for credentialing.

### Neurosurgeon involvement

In 5 centers, all cases must be reviewed at a multidisciplinary conference prior to radiosurgery treatment. Such conferences included representation by medical oncology, radiation oncology, and neurosurgery. At 3 centers, neurosurgeons are not required to be involved in cases in which there is no evidence of spine instability or spinal cord compression. The multidisciplinary approach of credentialed physicians would seem valuable for appropriate patient selection. Six institutions have more than one specialist trained and credentialed to perform spine radiosurgery. None of the centers currently require a “backup” physician and physicist for spine radiosurgery cases. In the single European center, that was initially the policy.

### Role of industry and professional organizations

Three institutions require physicians and medical physicists to attend corporate sponsored training. At the other 4 institutions, proctoring alone is considered to be sufficient. Two of these 4 institutions also require attendance at a non-corporate sponsored academic society radiosurgery course, as well. Six centers responded that industry should assist new users in the development of institutional credentialing, modelling that credentialing after the current requirements for Leksell Gamma Knife (Elekta AB, Stockholm, Sweden) training and credentialing.

All centers responded that spine radiosurgery education should be provided by both industry as well as by professional organizations. Corporate as well as non-corporate sponsored training were felt to be complimentary and both were considered important for training. Finally, all centers felt that national and international professional societies should be involved in the development of guidelines for requirements for spine radiosurgery credentialing.

### Perceptions regarding the importance of credentialing requirements

There was close agreement among all institutions that credentialing is essential for the development of a new spine radiosurgery program. It was felt that such credentialing would translate into improved patient clinical outcomes and especially patient safety. A formal credentialing process was also felt to be important for hospital management, especially to assist in a better understanding of staffing requirements for a spine radiosurgery program.

## Discussion

Often, the limiting dosimetric factor associated with conventional spine radiotherapy is the dose to the spinal cord. Conventional external beam radiotherapy lacks the precision to deliver large single-fraction doses of radiation to vertebral tumors near radiosensitive structures such as the spinal cord. Although short term pain control may suggest high rates of partial response, the complete response rates may be sub-optimal. Spine radiosurgery allows for the ability to better shape the radiation around the critical structures such as the spinal cord and effectively dose escalate while still maintaining safe dose limits to the spinal cord and other surrounding organs at risk (for example the esophagus, bowel, etc.). It is postulated that precise confinement of the radiation dose to the treatment area, as is the case for intracranial radiosurgery, should increase the likelihood of successful tumor control and clinical response at the same time that the risk of adjacent neurological injury is minimized [1-3,7,15,18,20,33-36]. It is this premise upon which the widespread adoption of radiosurgery for the treatment of spine and paraspinal tumors has been based.

With the increasingly widespread adoption of intensity-modulated radiotherapy (IMRT) combined with imaged guided techniques to allow for stereotactic body radiotherapy treatments, there has been increasing attention focused on the safety of these highly complex technologies. Initially, attention focused on the ability of centers to plan IMRT effectively. External audits of dosimetric comparisons between three-dimensional conformal radiotherapy (3D-CRT) and IMRT plans were performed and published [37]. While most clinical studies that demonstrated to yield superior conformality of the target volume and avoidance of critical structures in treatment plans were conducted in academic centers with rich experience in IMRT, it is less clear if these same dosimetric gains seen by IMRT over 3D-CRT can be seen at all centers. One major concern is the reproducibility of target volume delineation by clinicians who lack significant clinical experience. Radiation oncologists may not be formally trained and cognizant of the anatomy related to the spine and paraspinal structures [37]. One published external audit confirmed that a more experienced center is more able to maximize the dosimetric advantages of IMRT [37]. These authors went on to recommend that future efforts should be directed toward addressing this learning curve by establishing protocols, conducting educational workshops, and fostering institutional mentorship programs or communications to close the gap between experienced and less-experienced centers. The International Spine Radiosurgery Consortium has published consensus guidelines for target volume definition in spinal stereotactic radiosurgery for common clinical situations in an effort to decrease the potential for contouring variability among clinicians in this regard [38].

The American Society for Therapeutic Radiology and Oncology (ASTRO) and the American College of Radiology (ACR) have put forth guidelines for the performance of IMRT [39], IGRT [40] and SBRT [32]. These guidelines were published as an educational tool designed to assist practitioners in providing appropriate care of patients. The guidelines for SBRT specifically address the qualifications and responsibilities of personnel, including the radiation oncologist, medical physicist, radiation therapist, and “other participants” (e.g. surgeons). The authors state that strict protocols for QA must be followed. SBRT requires levels of precision and accuracy that surpass the requirements of conventionally fractionated radiation therapy or conventional IMRT. The SBRT process requires a coordinated team effort between the radiation oncologist, the medical physicist, the medical dosimetrist, and the radiation therapist. These inclusive guidelines also address issues of quality control and improvement, safety, infection control, patient education, documentation, and follow-up recommendations. A more recent scope of practice guideline was published by CARO that focused on spine, lung and liver SBRT. This document also clarified the role of the radiation oncologist, departmental considerations prior to implementation of an SBRT program and QA measures to be considered for a safe program. However, no recommendations are offered regarding the institutional credentialing of personnel involved in the delivery of SBRT [32]. The current manuscript focused on the training aspects of the credentialing process for spine radiosurgery. Our questionnaire did not address other phases of the credentialing process such as planning exercises and phantom testing such has been described elsewhere [29,30].

Several position statements have been published regarding quality assurance needs for modern image-based radiotherapy such as radiosurgery. Once such report summarized the consensus findings of a joint symposium of the ASTRO, the American Association of Physicists in Medicine (AAPM), and the National Cancer Institute (NCI) [27]. The recommendations from this body focused largely on the development of more appropriate proscriptive quality assurance tests (QA) for these newer SBRT delivery systems. Healthcare administrators need to assure the presence of appropriate personnel and ancillary equipment resources, as well as capital resources, when new advanced RT technology modalities are implemented. The pace of formalized clinical physics training must rapidly increase to provide an adequately trained physics workforce for advanced technology RT. Finally, government and private entities should support research directed toward addressing QA problems in image-guided RT therapies. While this consensus statement made specific recommendations regarding QA guidance for SBRT, they failed to address the issue of institutional training and credentialing of personnel.

Much of the initial focus for credentialing for SBRT related to quality assurance issues in conducting multi-institutional advanced technology clinical trials [29]. The first such work began in 2002 when the Radiation Therapy Oncology Group in North America began the process of developing multicenter prospective trials in lung cancer using SBRT [31]. These activities have had an impact not only on the treatment received by patients enrolled in clinical trials, but also on the quality of treatment administered to all patients treated in each institution. Such techniques have now been adopted globally [41].

Several cooperative groups have determined that the technologies used in certain clinical trials are sufficiently advanced to warrant specific credentialing of institutions that wish to participate in these trials [42]. The many challenges in credentialing institutions and participants for multi-institutional clinical trials in SBRT have been reviewed. The primary goal of credentialing is to reduce the deviation rate of data submitted to clinical trials. Credentialing offers a number of other benefits. Chief among these is the education of staff at the participating institution to ensure an understanding of the protocol and its goals. Cooperative groups have experienced deviation rates that sometimes amount to as much as 17% of the cases submitted [42]. A report by the National Cancer Institute Work Group on Radiotherapy Quality Assurance described the redesigning of radiotherapy quality assurance that included opportunities to develop an efficient, evidence-based system to support clinical trials [28]. The group made four recommendations for the improvement of multi-institutional clinical trial QA that might decrease these relatively high deviation rates.

While professional organizations and government sponsored agencies have published guidelines regarding credentialing for radiosurgery, these recommendations were once again within the context of multi-center government sponsored clinical trials. The National Cancer Institute-sponsored Advanced Technology Quality Assurance Consortium pioneered the development of an infrastructure and QA method for advanced technology clinical trials that requires volumetric digital data submission of a protocol patient’s treatment plan and verification data [30]. The quality assurance of imaged-guided radiation therapy (IGRT) within clinical trials is currently in its early stages, but its importance will continue to grow as IGRT becomes more widely adopted. The outcome of clinical trials may be affected by a known wide degree of variation in dose delivery of IMRT across multiple sites [25]. Compared to conventional radiotherapy, the steep dose gradients and tight margins achieved with IMRT have an even greater probability of leading to detrimental consequences via reduced tumor control and increased toxicity. The IGRT component of clinical trials that includes sophisticated planning and treatment protocols must undergo stringent QA. For this reason, IMRT QA is an essential part of the credentialing process for clinical trials.

The ultimate goals of the process of credentialing of personnel are for patient safety and quality of care. Areas of strong agreement summarizes the areas for which there is strong agreement among institutions for the process of spine radiosurgery credentialing. These areas might be considered the *minimum* requirements necessary for the safe and effective implementation of a new spine radiosurgery program. Areas of some agreement summaries the areas for which there is some agreement among institutions for the process of spine radiosurgery credentialing.

Quality outcomes are now being assessed by private payers and professional societies. These outcomes are also more readily available to patients. Differences in quality assessed through registry initiatives may drive the professional societies, health care institutions, or payers to mandate a more rigorous credentialing process for clinicians providing high technology services such as spinal radiosurgery. Relatively few studies have been undertaken to address the specific issue of credentialing of personnel within an individual institution. Njeh et al. supported that radiation departments should be certified to proven new technologies such as IGRT [43]. Radiosurgery has become part of formal neurosurgery resident education in the United States [44]. There are also calls for better coordination between surgeons and radiation oncologists in the cancer treatment decision-making process for spine and other primary tumor sites [45]. Furthermore, already there are published recommendations of conjoint statements by professional and governing organizations on credentialing and delineation of privileges for therapeutic procedures using radiopharmaceuticals [24]. To our knowledge, no such statements have been made regarding credentialing and delineation of privileges for extracranial radiosurgery, including spine radiosurgery.

The current survey study revealed that there is an important need for guidance regarding the credentialing of physicians and staff for the participation in the treatment of spine radiosurgery at an individual institution. Broad agreement exists among experienced spine radiosurgery centers in the areas of policies and procedures, training requirements, surgeon involvement, and the role of industry and professional organizations in the credentialing process. Strong agreement in the credentialing processes was observed despite the multi-national nature of the consortium, which consists of members from the United States, Canada and Germany. Furthermore, all institutions feel strongly regarding the importance of such credentialing in the development of a safe and effective spine radiosurgery program.

## Conclusions

Radiosurgery represents a great advance in the treatment of spine tumors, but is also dependent on the correct application of highly complex and advanced technologies involving a number of highly trained individuals from multiple disciplines. This represents the first multi-national report of the current practice of institutional credentialing for spine radiosurgery. Key methodologies for the safe implementation and credentialing of spine radiosurgery have been identified. There is strong agreement regarding the issues of policies and procedures, training requirements, neurosurgeon involvement, and the role of industry and professional organizations in the credentialing process. Finally, there is strong agreement among centers that credentialing is an important component of a safe and effective spine radiosurgery program. Such recommendations may serve as a useful reference for the implementation of a spine radiosurgery program. Furthermore, they might be considered the minimum requirements necessary for the implementation of a new spine radiosurgery program.

## Competing interests

This research was partially supported through an Elekta Stereotactic research grant with all institutions being members of the Elekta Spine Radiosurgery Research Consortium. This work and these data, however, are the intellectual property of the individual group members and their sponsoring institutions.

## Authors’ contributions

PG designed the study, collected the data and performed the data analysis. AS, JS, RK, SC, JF, MQ, DF, IG, JS, BW, KO, RS, and MG all participated in data collection and analysis. All authors performed critical review of the manuscript and finally approved the manuscript.
